# A New Generation of Cell Therapies Employing Regulatory T Cells (Treg) to Induce Immune Tolerance in Pediatric Transplantation

**DOI:** 10.3389/fped.2022.862807

**Published:** 2022-05-11

**Authors:** Esther Bernaldo-de-Quirós, Marjorie Pion, Marta Martínez-Bonet, Rafael Correa-Rocha

**Affiliations:** Laboratory of Immune-Regulation, Instituto de Investigación Sanitaria Gregorio Marañón (IiSGM), Madrid, Spain

**Keywords:** pediatric transplant, immune tolerance, Treg cells, cell therapy, graft rejection, thyTreg

## Abstract

Kidney transplantation is the most common solid organ transplant and the preferred treatment for pediatric patients with end-stage renal disease, but it is still not a definitive solution due to immune graft rejection. Regulatory T cells (Treg) and their control over effector T cells is a crucial and intrinsic tolerance mechanism in limiting excessive immune responses. In the case of transplants, Treg are important for the survival of the transplanted organ, and their dysregulation could increase the risk of rejection in transplanted children. Chronic immunosuppression to prevent rejection, for which Treg are especially sensitive, have a detrimental effect on Treg counts, decreasing the Treg/T-effector balance. Cell therapy with Treg cells is a promising approach to restore this imbalance, promoting tolerance and thus increasing graft survival. However, the strategies used to date that employ peripheral blood as a Treg source have shown limited efficacy. Moreover, it is not possible to use this approach in pediatric patients due to the limited volume of blood that can be extracted from children. Here, we outline our innovative strategy that employs the thymus removed during pediatric cardiac surgeries as a source of therapeutic Treg that could make this therapy accessible to transplanted children. The advantageous properties and the massive amount of Treg cells obtained from pediatric thymic tissue (thyTreg) opens a new possibility for Treg therapies to prevent rejection in pediatric kidney transplants. We are recruiting patients in a clinical trial to prevent rejection in heart-transplanted children through the infusion of autologous thyTreg cells (NCT04924491). If its efficacy is confirmed, thyTreg therapy may establish a new paradigm in preventing organ rejection in pediatric transplants, and their allogeneic use would extend its application to other solid organ transplantation.

## Introduction

Kidney transplantation is the treatment of choice for pediatric patients with end-stage renal disease. In children, congenital anomalies of the kidney and urinary tract and glomerular diseases are the most frequent indications for kidney transplantation (1). Although kidney transplantation is one of the significant milestones of modern medicine, immune allograft rejection remains the main obstacle to definitive successful transplants. According to the latest update of the OPTN/SRTR Kidney Annual Report, the incidence of acute rejection within the first year for pediatric kidney transplants is 11.1% (2), and the occurrence of acute rejection episodes is associated with poorer survival of the graft along the time. Besides, chronic rejection is one of the leading causes of graft loss in children. Therefore, a countdown is settled as soon as a patient receives a transplant that is hallmarked by the slow degeneration of the organ due to rejection, giving graft survival rates of 12–15 years in kidney transplanted children (3). While in adult patients, the transplanted organ can extend the patient's lifetime to values close to the life expectancy of a healthy subject (80 years), when it comes to pediatric recipients, the viability of transplanted organs is very far from guaranteeing the average life expectancy.

The existing scientific evidence supports that only the induction of immunological tolerance, re-educating the recipient's immune responses, will allow the indefinite survival of the graft. In this sense, one of the most promising alternatives to increase the life expectancy of transplant patients is to induce tolerance through cellular immunotherapy without using pharmacological immunosuppression, thus eliminating the toxic effects of these therapies and maintaining a competent immune system (4). Along these lines, different approaches to cell therapy have been explored, being regulatory T cells (Treg), the cells with the most significant potential and most studied for this purpose. In this review, we will summarize the current knowledge about Treg role to prevent graft rejection and emphasize the state of Treg cell-based therapies in solid organ transplantation, along with their obstacles and limitations surrounding their access to the clinic in the pediatric setting. Last, we will also report an innovative approach that has been developed in our group that makes this Treg therapy accessible to transplanted children.

## Mechanisms of Treg Suppressive Function and Transplant Tolerance

There are many cell types involved in the preservation of tolerance, both of innate and adaptive immunity, such as tolerogenic dendritic cells (DC) (5), tolerogenic monocytes or macrophages (6), tolerogenic natural killer (NK) cells (7) and regulatory B cells (8); together with mesenchymal stem cells (9). The most studied are Treg cells, a subpopulation of CD4+ T cells with suppressive capacity that plays a fundamental role in regulating the immune processes intrinsic to graft acceptance. Furthermore, an additional role in tissue repair and regeneration has also been described recently for these cells (10–12).

Tregs are characterized by the high and stable expression of the α chain of the IL-2 receptor (CD25) and the transcription factor Foxp3 (forkhead box protein 3), which is critical for the development and maintenance of the function and the Treg phenotype (13). Tregs can suppress the effector function of a wide range of cells, including CD4+ and CD8+ T cells, B cells, DCs, macrophages, granulocytes, NK cells, and osteoclasts (14). For that, Treg cells can produce anti-inflammatory cytokines (IL-10, IL-35 and TGF-β) that directly inhibit effector T cells (Teff). They can also release perforin and granzyme that damage the target-cell membrane and cause apoptosis. Due to the high expression of CD25, Tregs can “sequester” IL-2, decreasing IL-2 reserves in the microenvironment and thereby reducing the proliferation of Teff and NK cells, as well as their effector function. Tregs have been shown to directly affect B cells through the PD-L1/PD-1 interaction and on DC through CTLA-4 (cytotoxic T lymphocyte antigen 4) and LAG-3 (lymphocyte activation gene 3). The expression of CD39 and CD73 ectoenzymes on Tregs mediate the conversion of ATP (pro-inflammatory signal) to adenosine (anti-inflammatory mediator) and AMP, indirectly reducing the proliferation of Teff. Tregs can also induce monocytes toward anti-inflammatory M2 macrophages and prevent their differentiation to pro-inflammatory M1 macrophages (15).

In animal models, Treg cells have demonstrated their ability to delay/prevent graft rejection and promote transplant tolerance and indefinite graft survival (16–19). Specifically, it has been observed that adoptive transfer of Treg cells in combination with a short treatment of calcineurin inhibitor (CNI) prevent acute rejection and induce long-term graft survival in a rat kidney transplant model (19). Moreover, some studies, the majority of them in the context of liver transplantation, carried out in adult and pediatric transplanted individuals with operative tolerance, showed a significant increase in the number of circulating Tregs and in graft infiltrates compared to non-tolerant individuals (20–23). On the contrary, a Treg deficiency could seriously compromise the main peripheral tolerance mechanisms, and indeed, decreased Treg values have been associated with acute rejection and chronic allograft nephropathy in kidney transplant recipients (24–27). It has been described that the relationship between Treg cells and Teff cells is crucial for the induction of tolerance or the development of graft rejection responses (28–30).

## Immunosuppressive Drugs and their Impact on Treg

Immunosuppressive treatment of transplant patients aims to limit the recipient's immune response against the graft. The treatment begins in the perioperative period, continues after transplantation with high levels of immunosuppressants, and later, the doses can be reduced to a maintenance therapy administered throughout the patient's life. There is a wide variety of immunosuppressive drugs available that allow the establishment of different treatment combinations that vary according to the transplanted organ, the patient, the risk of rejection, and also between countries and institutions (31). In most cases, kidney transplanted children receive some induction therapy with basiliximab (BXM) or thymoglobulin (ATG) and maintenance therapy with tacrolimus (TAC), mycophenolate (MMF), and corticosteroids (Pred), followed by TAC and MMF with Pred cessation (2).

The immunosuppressive regimen could affect the Treg cell population, essential to preserve immune tolerance after transplantation. BXM is a monoclonal antibody that binds to the IL-2 receptor (CD25), completely blocking the interactions between CD25 and IL-2. Its use aims to block IL-2 uptake by activated Teff, harmful in the context of transplantation, thus preventing their proliferation (32). However, Treg cells also express high levels of CD25 constitutively and can also be affected by this drug. Indeed, Treg cells are extremely dependent on IL-2 and the transduction of intracellular signals related to the IL-2 receptor (33). Although other cytokines can replace the function of IL-2 in conventional T cells, IL-2 is essential for Treg development, homeostasis, and function (34). Moreover, since they do not produce IL-2, they are dependent on IL-2 secreted by other T cells and dendritic cells. We have shown that the induction therapy with BXM has an apparent detrimental effect on Treg cells values, which could negatively affect the protective role of Treg in transplanted children in the period of the highest incidence of acute rejection (35). Regarding the immunosuppressive drugs used as maintenance therapy, TAC is a CNI drug that directly affects Tregs' activation, proliferation and survival, but it can also indirectly affect Tregs by limiting IL-2 production by Teff (33). Long-term and high doses of CNI have been related to low Treg numbers, weaker Treg function and phenotype in children with stable liver and kidney allografts (36). Concerning the MMF, its effect on the Tregs remains controversial. On one hand, *in vitro* experiments indicate that MMF does not alter the phenotype of Tregs or can even promote the predominance of Tregs on Th17 cells (33); and it has also been observed that kidney transplant patients receiving MMF showed higher Treg levels than those treated with everolimus (37). However, on the other hand, in animal models that received cell therapy with Tregs, the administration of MMF reduced the efficacy of Tregs in preventing rejection (38). Finally, corticosteroids appear to benefit the prevalence of Tregs and their activity by facilitating TGF-β signaling and Foxp3 expression (33). In the case of heart-transplanted children who are thymectomized before or during the transplantation procedure, we demonstrated that the combination of thymectomy, which is the source of new Treg cells, with the administration of immunosuppressive drugs (TAC, MMF, and Pred) produce a profound immune dysregulation. It is characterized by a decrease in Treg cell counts and an imbalance between Treg cells and Teff cells, which could compromise the natural or intrinsic tolerance mechanisms (39).

Considering the evidence supporting the role of Tregs in preventing rejection in transplanted children, monitoring of Treg and Teff cell values and the imbalance between both cell subsets could constitute markers of early risk of rejection that can help to avoid graft damage before it occurs. New strategies aimed at preventing the decrease in Treg or restoring their frequencies could impact the prevention of graft rejection. Interventions such as: (i) the choice of immunosuppressive drugs with less impact on Tregs; (ii) the supplementation with vitamin D, which has been shown to increase Treg survival (40) and reduce the risk of acute rejection (41, 42); or (iii) even a cellular therapy with Treg cells (43, 44) could counteract the imbalance between Treg and Teff cells.

## Strategies to Provide Indefinite Graft Survival: Cellular Therapies With Treg

One of the most promising alternatives to provide indefinite graft survival and thus increase the life expectancy of transplanted patients without morbidity is to induce immune tolerance by cellular immunotherapy. Establishing tolerance in transplantation aims to deliberately abate the detrimental allograft-specific immune response while avoiding the toxicity of long-term immunosuppression and maintaining a competent immune system (4).

In the search for this “Holy Grail” of transplantation tolerance, all efforts are currently directed toward developing new immunomodulatory approaches with advanced cellular therapies mainly based on immune regulatory or suppressive cells to reduce or eliminate immunosuppressants. Among them, different cell therapy approaches are being explored, such as mesenchymal stem cells (9, 45, 46) and their exosomes (47), tolerogenic dendritic cells, myeloid-derived suppressor cells (MDSC), specifically regulatory macrophages. However, Treg cells, which are “professional” cells in terms of regulatory function and have been shown to play a crucial role in the prevention of immune rejection, could be the strategy with the greatest potential. Moreover, the tolerance mediated by these cells is highly antigen-specific and localized (48), being capable of migrating to sites of inflammation where they exert their suppressing function (49).

Treg therapies are generally based on Tregs expansion *ex vivo* followed by the re-infusion of expanded or induced Treg. However, others consider the possibility to expand them *in vivo* to limit the *ex vivo* cellular manipulation. Some preclinical models showed that activating a tumor necrosis factor receptor superfamily member 25 (TNFRSF25) with or without IL-2 could reduce the graft-vs.-host disease or prevent allergic lung inflammation in mice recipients (50). The antibody-mediated agonistic stimulation of TNFRSF25 was also shown to prolonge islets' allograft transplantation survival (51), which opens the possibility that the *in vivo* Treg expansion could contribute to reaching immunological tolerance in organ transplantation. Nevertheless, this possibility can be envisaged only in patients with slight or no Treg dysfunction.

The therapeutic efficacy of Treg cell transfer in the context of solid organ transplantation has been demonstrated in preclinical studies with animal models. Tsang et al. reported that Treg infusion combined with temporary CD8+ T cell depletion and a short course of rapamycin-induced indefinite graft survival in an animal model of heart transplantation (52). Furthermore, in kidney transplantation models employing non-human primates, *ex vivo* expanded Treg therapy has been shown to prolong graft survival and prevent acute rejection (53–55). In the study by Duran-Struuck et al. monkeys that received a bone marrow transplant together with the infusion of polyclonal Treg cells (from the same donor) were able to accept a kidney graft without immunosuppression for more than 294 days, in comparison with recipients not receiving Treg therapy rejecting transplanted kidneys at 21–28 days (55).

Along with the experimentation carried out in non-human primates, humanized animal models are an additional proof of concept that Treg therapy has a suppressive effect on the immune system, favoring tolerance of the transplanted organ. Studies carried out in these animals have shown that mice that receive only allogeneic peripheral blood mononuclear cells (PBMCs) reject human skin grafts, while those that also receive Tregs show stable survival of human skin transplantation together with a reduction of human CD8+ T cells in the infiltrates of the skin graft (56). In the work of Wu et al. Treg therapy prolonged survival of pancreatic islet transplantation in a humanized diabetic mouse model, resulting in accumulation of Treg in lymph nodes and suppression of both proliferation and IFN-γ production by T cells (57). Furthermore, the potential of Treg cells has also been identified in the study by Nadig et al. in a clinically relevant humanized mouse model by preventing the development of atherosclerosis of the transplanted organ, which is the hallmark of chronic graft dysfunction (58). Ultimately, these preclinical models reflect the potential efficacy of a therapy based on the *ex vivo* expansion of Treg cells and subsequent re-infusion to achieve indefinite graft survival in transplant patients.

After animal models, the safety and potential efficacy of the Treg therapy to re-establish the immune tolerance is being evaluated in clinical trials in adult transplanted patients. Currently, ongoing clinical trials are using a therapy with Treg cells obtained from peripheral blood to prevent rejection of the transplanted organ in adult patients, most of them in the context of kidney and liver transplantation [reviewed in (15, 43, 59, 60)]. However, few definitive conclusions about these studies have been published, and most of them are still in phase I or phase I-II. The main published results, referring to phase I or phase I-II clinical trials employing Treg cells in the context of adult kidney transplantation are: the TASK trial (61), the TRACT trial (62) and the “The ONE Study” consortium (63–65).

In 2017, the results of the TASK trial (NCT02711826) conducted at the University of California (UCSF) (61) were published. Three kidney transplant patients were treated with *ex vivo* expanded autologous Tregs using a medium rich in deuterated glucose to label and track cells *in vivo*. Patients received a single infusion of 320 × 10^6^ and maintained their immunosuppressive regimen with TAC, MMF and Pred. The infused Tregs peaked in circulation the first week after infusion, with detectable signals during the first month falling at 3 months after infusion. None of the patients presented infusion reactions, and no infections or malignancies were observed in the 1-year follow-up period.

The results of the TRACT trial (NCT02145325) were published in 2018 by Mathew et al. from the Northwestern University, Chicago, including a total of 9 kidney transplant patients that were divided into three groups of 3 patients who received 0.5, 1, and 5 × 10^9^ *ex vivo* expanded autologous Treg cells at 60 days post-transplantation (62). In this phase I safety trial, the number of infused Tregs increased in the periphery, and no therapy-related adverse events, infections, or rejection events were observed up to 2 years post-transplantation.

The “The ONE Study” is an international consortium that involves eight institutions in five European countries and the US, investigating the safety and feasibility of different regulatory cell populations (Treg, tolerogenic DC and regulatory macrophages) in living donor kidney transplant patients (63). Two autologous polyclonal Treg cell products (pTreg-1 and pTreg-2) and two donor antigen-specific Treg products were used. All products ranged from 0.5 to 10 × 10^6^ cells/kg of patient's weight and were administered in a single infusion 10 days post-transplantation. Patients were routinely monitored for the primary endpoint biopsy-confirmed acute rejection (BCAR) within 60 weeks post-transplantation. The results were published in mid-2020 and showed good safety data. However, the efficacy results are not entirely conclusive, as BCAR rates were comparable between the standard immunosuppressive and cell therapy groups (12 vs. 16%). Subsequently, results from two more clinical trials within the ONE Study consortium have been published. The ONEnTreg13 phase I-IIa trial (NCT02371434) conducted at the Charité-University Hospital in Berlin compared 11 living donor kidney transplant recipients who received the autologous Treg therapy with 9 patients from the reference group (64). Tregs were infused seven days after kidney transplantation at escalating doses of 0.5, 1.0, 2.5–3 × 10^6^ cells/kg of the patient, with no dose-related toxicity observed. Same 100% 3-year allograft survival was observed in both groups. Importantly, in eight of 11 patients receiving the Treg therapy, it was possible to achieve lower TAC's monotherapy dose compared to the reference group that remained on standard dual or triple immunosuppression (TAC, MMF and Pred). The other published clinical trial part of The ONE Study consortium was conducted in the United Kingdom as a phase I trial, including 12 living donor kidney transplant recipients divided into four groups receiving 1, 3, 6, or 10 × 10^6^ Treg/kg at 5 days post-transplantation, and 19 patients in the reference group (65). No safety concerns were observed, and in 4 patients who received the Treg therapy, MMF was withdrawn, remaining on TAC monotherapy.

A possible risk of Treg therapy could be associated with the fact that high numbers of Treg cells can suppress antitumor responses favoring the tumor progression (66). Indeed, higher numbers of Treg have increased the odds of cutaneous squamous cell carcinoma appearance in kidney transplant recipients (67). Nevertheless, no clinical trial employing Treg in kidney transplanted adults has reported a higher incidence of cancer in treated patients (61–65). Although we will have to wait for more long-term results in this clinical trial and other ongoing clinical trials to have conclusive evidence, the data so far indicate that Treg therapy in the context of solid organ transplantation is safe. The infused cells are well-tolerated by patients, even when administered at high doses. Many questions in terms of efficacy remain unanswered regarding the Treg doses, the timing of infusion, the Treg survival, the *in vivo* Treg's mode of action as their location. However, the clinical efficacy results are not entirely encouraging, presenting a low/short therapeutic effect that would be not enough to provide the indefinite survival of the graft.

## Limitations of Current Approaches of Treg Cell Therapy to Prevent Graft Rejection

The current Treg therapy strategy used by nearly all trials in the context of solid organ transplantation consists of: (a) drawing peripheral blood from the patient; (b) purification of Tregs from peripheral blood; (c) *ex vivo* activation/expansion of Tregs to obtain an appropriate number of cells; and (d) infusion of Tregs obtained in the patient himself (autologous use) (61–63, 68, 69). However, this widely used strategy presents a series of limitations regarding the source and the method used to obtain the Treg cells that have compromised its effectiveness.

The frequency of Tregs in peripheral blood accounts for only 5–10% of total CD4+ T cells (70). Because Tregs are isolated from peripheral blood based on the exclusive expression of the surface markers CD4 and CD25, the final product could contain a significant percentage of activated Teff that also express these two markers. Therefore, to gain purity, it is necessary to use additional markers such as CD127 (71), reducing cell yield and adding complexity to the good manufacturing practice (GMP) protocol. Taking as reference the data reported by Balcerek et al. in none of the 41 therapeutic Tregs, when isolated from peripheral blood, was possible to obtain more than 11.8 × 10^6^ Tregs (72). For this reason, it is necessary to carry out a massive *ex vivo* expansion to obtain clinically adequate yields.

The quality of Treg cells is closely dependent on the phenotype or activation/differentiation state of the cells. Most of the Tregs purified from adults have a memory and more differentiated phenotype that limits their functionality. As Miyara et al. demonstrate, Treg cells with a memory phenotype (CD45RA–) can lose their Treg phenotype due to presenting more methylated *Foxp3* gene regions, so their suppressive capacity is more limited (73). Furthermore, the limited quality of adult Tregs is further worsened when they are exhaustively expanded to reach a sufficient number for their therapeutical use. The work of Hoffmann et al. reported that repeated expansion of Treg cells causes a marked loss of suppressive capacity and even the conversion of Treg into Teff, posing an added risk for the occurrence of rejection (74, 75).

Some studies consider the population of naïve Treg cells (CD45RA+) as the optimal subpopulation for therapy since these cells have more remarkable survival and maintain their suppressive capacity and phenotype stability (76, 77). While Treg cells with memory phenotype stimulated and expanded *ex vivo* may lose Foxp3 expression and its Treg phenotype, Treg cells with naïve phenotype can maintain Foxp3 expression and suppressive capacity after repeated stimulation and expansion (74). In pediatric patients, it is expected that Treg cells could present a more naïve phenotype with more remarkable survival and functional capacity compared to Tregs from adult patients.

Despite the high quality of pediatric Tregs, no clinical trials have been performed with Treg in transplanted children to prevent rejection. The usual strategy of isolating Treg from peripheral blood is not feasible in pediatric patients, especially in their shortage, since large volumes of blood cannot be drawn due to their low weight (78). Thus, the amount of Tregs recovered from peripheral blood would be even more restrictive than in adults. As an example, in a 13 kg child, the maximum volume of blood that can be safely extracted would be around 50 ml. Taking into account that Treg represent 5% of the total blood volume, the available amount of CD45RA+ Treg would be minimal, as indicated in (78). Based on the reference values reported in the study by Schatorjé et al. (79), the average Treg count for a 2-year-old child of that weight is 120,000 cells per ml. Therefore, the theoretical Treg number that could be obtained from 50 ml of blood would be 6.24 × 10^6^ Tregs. However, since the yield in cellular isolation is <20% of the theoretical number (16.2%) (72), only 1 × 10^6^ Tregs would be isolated, being clearly insufficient to administer a single therapeutic dose. This issue could be solved by doing exhaustive *ex vivo* expansion cycles, but as mentioned, this expansion would lead to Treg differentiation, losing the advantage of their more naïve or undifferentiated phenotype.

Another strategy that has been used with some success is to obtain Tregs from umbilical cord blood, which shares the advantage of being mostly CD45RA+ naïve cells (80), but also has the important limitation in the number of cells. As described by Riley et al., from a cord blood unit, between 5 and 7 × 10^6^ Tregs can be obtained (81), which would still be an insufficient number for a therapeutic dose and therefore requires numerous rounds of expansion. In some trials, those cells were expanded up to 27,000 times to achieve a single therapeutic dose (82). Despite the limited number of cells available, the potential efficacy of umbilical cord blood Tregs has been demonstrated when used as an allogeneic therapy to prevent graft vs. host disease (GVHD) in adults (82, 83). However, so far to our understanding, cord blood Tregs have not been used in the context of solid organ transplantation.

## Thymic Tissue As a New Source of Treg: An Innovative Approach That Could Make Treg Cell Therapy Accessible To Transplanted Children

Our group decided to explore a new strategy to obtain an adequate quantity of Treg cells suitable for cellular therapy from an alternative source to peripheral or cord blood that could be transferred to the pediatric setting. With this aim, we studied whether the thymus that is routinely removed and discarded in pediatric cardiac surgeries could be a source of therapeutic Treg cells. The thymus is the organ where Treg cells are produced, like the rest of T lymphocytes. Thus, Tregs obtained from this tissue are immature and have an undifferentiated phenotype (84). They also present a greater suppressive capacity and, above all, greater stability of the Treg-related phenotype. Dijke et al. previously reported the potential of discarded human thymuses as an excellent source of Treg to overcome the limitations of peripheral blood in terms of yield, stability, suppressive capacity and survival (85). Indeed, we have developed a novel GMP-protocol to obtain massive amounts of highly pure, suppressive and stable Treg from thymuses routinely discarded during pediatric cardiac surgeries (thyTreg) after a 7-days culture; being our thyTreg product already approved by the Spanish Drug Agency (AEMPS) to be administered as cell therapy (86).

The strategy of using the thymus as a source of Treg was of particular interest in treating children with heart transplants since, during the transplant, surgeons are forced to remove the thymus, or part of it, in order to gain access to the heart. Thus, the thymic tissue would be available in these patients at the moment of the transplant intervention, and it would allow the generation of autologous cell therapy. In 2020, we initiated a pioneering phase I-IIa clinical trial to prevent rejection in heart-transplanted children through the infusion of autologous thyTreg cells (NCT04924491). This clinical trial represents the first Treg cell therapy performed in transplanted children and, importantly, the first worldwide employing thyTreg from thymic tissue. In addition, the large number of thyTreg cells obtained with this strategy opens the possibility for the administration of successive doses at different time intervals or when signs of rejection appear to induce graft tolerance. If its efficacy is confirmed, the thyTreg therapy may establish a new paradigm in preventing organ rejection in solid organ transplantation. At present, we are also exploring the allogeneic use of thyTreg, which would allow us to extend the use of this therapy to kidney-transplanted children or children transplanted with other organs.

## Conclusions

Treg cells play a crucial role in the balance between immune tolerance and graft rejection. Immunosuppressive drugs addressed to limit the immune responses against the graft could also impair the intrinsic tolerance mechanism promoted by Tregs. Restoring the balance between Treg and Teff cells by increasing Treg reserves through cell therapy can be an effective strategy to induce immune tolerance and prevent graft rejection. The ongoing clinical trials employing Treg therapy to prevent solid organ rejection in adults use autologous Treg cells obtained from peripheral blood. However, this strategy cannot be employed in transplanted children due to the limited number of Treg cells available in peripheral blood. The therapeutic strategy developed in our group that employs thyTreg purified from pediatric thymic tissue represents an innovative approach that could make Treg therapy available in transplanted children. From here, a wide range of possibilities opens up to exploit the potential of this therapeutic arsenal that constitutes the thyTreg in the prevention of organ rejection and the treatment of other diseases associated with phenomena of hyperactivation of the immune system.

## Author Contributions

EB-d-Q, MP, MM-B, and RC-R have organized and discussed the review and contributed to the manuscript's elaboration. All authors have participated sufficiently in this work to take public responsibility for the content. All authors contributed to the article and approved the submitted version.

## Funding

This work was supported by grants from Fundación Familia Alonso (FFA- FIBHGM 2019), Instituto de Salud Carlos III (ISCIII) cofinanced by FEDER funds (ICI20/00063; PI18/00495; PI18/00506, PI21/00189). EB-d-Q was supported by a grant from Comunidad de Madrid (EXOHEP-CM. B2017/BMD3727). MM-B was supported by the Sara Borrell Program from ISCIII (CD18/00105) and Marie Skłodowska-Curie Actions (H2020-MSCA-IF-2020- 101028834).

## Conflict of Interest

The authors declare that the research was conducted in the absence of any commercial or financial relationships that could be construed as a potential conflict of interest.

## Publisher's Note

All claims expressed in this article are solely those of the authors and do not necessarily represent those of their affiliated organizations, or those of the publisher, the editors and the reviewers. Any product that may be evaluated in this article, or claim that may be made by its manufacturer, is not guaranteed or endorsed by the publisher.
